# Enhancing Primary Care and Mental Health Integration for Women Veterans with Complex Healthcare Needs Using Evidence-Based Quality Improvement

**DOI:** 10.1007/s11606-024-08737-3

**Published:** 2024-04-30

**Authors:** Kimberly S. Clair, Elizabeth M. Yano, Jacqueline J. Fickel, Julian Brunner, Ismelda Canelo, Alison Hamilton

**Affiliations:** 1 HSR&D Center for the Study of Healthcare Innovation, Implementation, and Policy, VA Greater Los Angeles Healthcare System, Los Angeles, CA USA; 2 Department of Health Policy and Management, Fielding School of Public Health at University of California Los Angeles, Los Angeles, CA USA; 3 Department of Psychiatry and Biobehavioral Sciences, David Geffen School of Medicine at University of California Los Angeles, Los Angeles, CA USA

**Keywords:** women’s health, primary care, mental health, quality improvement.

## Abstract

**Background:**

Women Veterans with co-morbid medical and mental health conditions face persistent barriers accessing high-quality health care. Evidence-based quality improvement (EBQI) offers a systematic approach to implementing new care models that can address care gaps for women Veterans.

**Objective:**

This study examines factors associated with the successful deployment of EBQI within integrated health systems to improve primary care for women Veterans with complex mental health needs.

**Design:**

Following a 12-site (8 EBQI, 4 control) cluster randomized study to evaluate EBQI effectiveness, we conducted an in-depth case study analysis of one women’s health clinic that used EBQI to improve integrated primary care–mental health services for women Veterans.

**Participants:**

Our study sample included providers, program managers, and clinic staff at a women Veteran’s health clinic that, at the time of the study, had one Primary Care and Mental Health Integration team and one women’s health primary care provider serving 800 women. We analyzed interviews conducted 12 months, 24 months, and 4 years post-implementation and call summaries between the clinic and support team.

**Main Measures:**

We conducted qualitative thematic analysis of interview and call summary data to identify EBQI elements, clinic characteristics, and reported challenges and successes within project development and execution.

**Key Results:**

The clinic harnessed core EBQI elements (multi-level stakeholder engagement, data-driven progress-monitoring, PDSA cycles, sharing results) to accomplish pre-defined project goals, strengthen inter-disciplinary partnerships, and bolster team confidence. Clinic characteristics that facilitated implementation success included prior QI experience and an organizational culture responsive to innovation, while lack of pre-existing guidelines and limited access to centralized databases posed implementation challenges.

**Conclusions:**

Successful practice transformation emerges through the interaction of evidence-based methods and site-specific characteristics. Examining how clinic characteristics support or impede EBQI adaptation can facilitate efforts to improve care within integrated health systems.

## BACKGROUND

Women Veterans are the fastest growing proportion of new Veterans Health Administration (VHA) users and have distinct healthcare needs.^1^ Women VHA users have high rates of anxiety^2^ and posttraumatic stress disorder (PTSD),^3^ and are more likely than men Veterans to be diagnosed with depression.^4,5^ Compared to men Veterans, women VHA users also have higher rates of military sexual trauma (MST),^6,7^ which is associated with a range of physical and behavioral health sequelae,^8^ including obesity,^9^ insomnia,^10^ chronic pain,^11^ and substance use disorders.^12,13^ The number of women Veterans who use specialty mental health services has grown five-fold over the past two decades,^1^ suggesting the need for evidence-based approaches to delivering high-quality integrated primary and mental health care services.

In 2010, the VHA developed guidance to provide comprehensive gender-sensitive primary care for women Veterans, including creating Women’s Health Patient Aligned Care Teams, or WH-PACTs.^14^ Although PACT has been associated with higher patient satisfaction and quality of care,^15^ less is known about how to tailor PACT to meet the needs of women Veterans. Ensuring provider proficiency in addressing gender-specific issues, increasing visibility of women Veterans’ care needs, and transforming care environments to promote women Veterans’ safety and dignity remain key challenges in the delivery of comprehensive gender-sensitive care within the VA.^16–18^

Evidence-based quality improvement (EBQI) offers a systematic approach to implementing new care models through multi-level partnerships among organizational leaders, local quality improvement (QI) teams, and researchers.^17^ Throughout implementation, EBQI promotes the use of “Plan-Do-Study-Act” (PDSA) cycles,^19^ formative data feedback, and periodic cross-site trainings and sharing of lessons learned to support spread of best practices.^17^ EBQI is highly flexible and may take different forms depending on the levels of leadership involved in priority setting—whether at the level of VHA service regions, or Veterans Integrated Service Networks (VISNs),^17^ or local VA medical centers (VAMCs)^20^—the focus of the implementation,^21^ and whether implementation occurs within a Practice-Based Research Network (PBRN),^22^ among other factors. While EBQI has demonstrated success improving uptake of evidence-based collaborative care practices,^23^ supportive employment,^24^ and PACT,^25^ few studies have investigated how EBQI may be harnessed to improve gender-sensitive primary care within integrated health systems. Further, although EBQI is typically evaluated through multi-site comparative studies of effectiveness or feasibility,^21,25–27^ its application on the ground can vary substantially, not only across sites but at different phases of implementation within the same organization.^28,29^ An in-depth examination of EBQI implementation at one site—from project development to completion—is useful for capturing the dynamic processes of change and improvisation that characterize EBQI implementation in real-time.

## OBJECTIVE

Our case study examines the development, implementation, and reported impact of a VA women’s health clinic’s locally prioritized EBQI activities to improve primary care for women Veterans with complex mental health needs. In this particular deployment of EBQI, panel methods were used to arrive at consensus on QI roadmaps of priorities that local VAMCs could choose from based on their local context and needs. For the VISN in this study, improving primary care for women Veterans with complex mental health needs was a QI roadmap priority that also aligned with local organizational and provider needs. We selected a case study approach to assess staff and provider perspectives at multiple timepoints throughout EBQI implementation to identify unexpected challenges and critical moments that fostered or impeded QI success.^30^ Our study examines how the clinic applied core features of EBQI to improve services for women Veterans with acute mental health needs. Our findings may prove valuable for facilities engaged in QI efforts to enhance women Veterans’ experiences with integrated primary-mental health services and QI efforts more broadly within integrated healthcare systems.

## DESIGN

Data are drawn from a cluster randomized trial that evaluated the effectiveness of EBQI for tailoring PACT to meet the needs of women Veterans.^17^ This study examines one intervention site—the women’s clinic at the Louis A. Johnson VAMC in Clarksburg, West Virginia—that developed and completed two EBQI projects with researchers at the VA Greater Los Angeles Healthcare System, who provided EBQI training, practice facilitation, formative data feedback, and technical workgroups for additional evidence-based support in priority areas.^17^ We selected this site for case study analysis based on their EBQI project aim: to enhance mental health services for women Veterans in comprehensive women’s health primary care teams.

We conducted secondary analysis of semi-structured interviews and call summaries with Clarksburg women’s clinic participants that were conducted during the cluster randomized trial at 12 and 24 months following implementation. The parent study design and methods are described elsewhere.^34^ Our primary data included telephone interviews with Clarksburg women’s clinic participants conducted 4 years post-implementation. Interviews were conducted by the lead author (KC) and lasted 45–60 min. Interviewees were asked to describe the process of developing an EBQI project focused on patients with acute mental health needs, their experiences and observations of clinic practices before and after introducing the EBQI project, challenges encountered, and reflections on the project’s long-term impact. All procedures were reviewed and approved by the VHA and UCLA Institutional Review Boards.

## PARTICIPANTS

Participants from the parent study include Clarksburg women’s clinic providers, program managers, and staff: a 12-month follow-up group interview was conducted with the Clarksburg Women Veteran Program Manager (WVPM), two licensed nurse practitioners, a certified registered nurse practitioner, and a health administrator, while a 24-month interview was conducted with the clinic’s EBQI team champion. Four-year follow-up interviews were conducted with the team champion and the Clarksburg WVPM.

The Clarksburg VAMC includes four primary clinics and serves approximately 1200 women Veterans, 500 of whom are patients of the Civilian Health and Medical Program of the Department of Veterans Affairs (CHAMPVA).^32^ In addition to TRICARE, a health care program for active-duty and retired service members and their families, the VA offers CHAMPVA, a cost-sharing comprehensive health care program for surviving spouses or children of Veterans with a service-connected death or disability. CHAMPVA beneficiaries are eligible for most healthcare services and supplies, including outpatient, inpatient, mental health, and emergency care, as well as family planning and maternity services.^32^ As a Model 3 women’s health center, the Clarksburg WHC has a separate entrance from the main hospital and private waiting room to provide coordinated, comprehensive primary care to women Veterans^33^ and co-located gynecological care, with clinic space and support staff shared with a full-time gynecologist and an in-house mammography clinic. The clinic had a Primary Care-Mental Health Integration (PCMHI) team and one women’s health primary care provider whose panel consisted of approximately 800 women; however, Mental Health and Primary Care services were not physically co-located at the facility.

## MAIN MEASURES

We analyzed secondary data, including a mid-point group interview with Clarksburg women’s clinic providers, program managers, and staff (*n* = 5, May 2016); a 24-month follow-up interview with the clinic’s EBQI team champion (June 2017); and call summaries between the Clarksburg EBQI team and the research team (October 2014–April 2016), which included meeting notes and action items. Primary data include a phone interview with the team champion (August 2020) and a phone interview with the Clarksburg WVPM (August 2020) who led the clinic’s EBQI project but had transitioned out of the WVPM role at the time of the interview.

The qualitative team (KC, JF) coded interview transcripts and call summaries to characterize key phases within the project’s development and execution, challenges encountered, responses to challenges, and measured or perceived project outcomes. Next, the first author (KC) identified elements of EBQI adopted throughout implementation as well as site-specific characteristics that facilitated or impeded EBQI activities. These elements were reviewed and validated (AH, EY). Our classification of “core EBQI elements” draws from previous research examining the successful EBQI-PACT implementation.^20,36^

## KEY RESULTS

**Figure 1:**
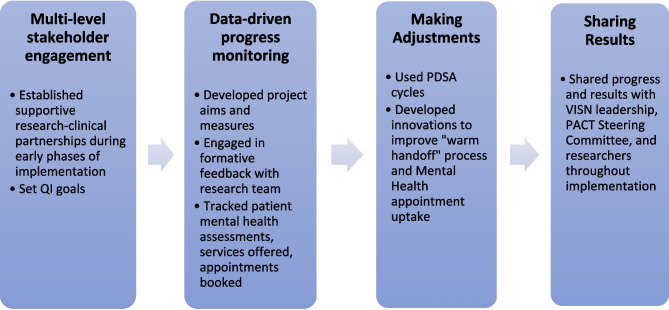
EBQI core features.

The Clarksburg WHC harnessed core features of EBQI to improve the quality of health services for women Veterans with complex mental health needs. These features include multi-level stakeholder engagement during the early phases of project development, data-driven progress-monitoring, the use of PDSA cycles, and sharing results both internally and with multiple levels of leadership (Fig. 1). Participant experiences with EBQI implementation, including barriers and facilitators, are described in Table 1. EBQI features were adapted to align with site-specific strengths and limitations in ways that either facilitated or impeded EBQI success at critical moments of implementation (Table 2).

**Table 1:** Participant Experiences with Site-Specific Barriers and Facilitators to EBQI Implementation

Facilitators	
Early support from research facilitators	“Normally…if you wanted to do anything there was really no structure in when we were going to meet, and setting up those regular meetings with the research team…we started making it a part of what are we doing for improving with these projects.”
Prior QI experience	“I think it just gave us that avenue to say, ‘You know, here’s a project, we got it accomplished, what’s our next problem?’”
Information-sharing sessions	“What made this successful was bringing us all together from different disciplines…we all had the same goals but once we were all in the same room, once everybody knew what was expected, it took off.”
Support from leadership	“We have had such a fabulous support from administration and some of the other teams…We didn’t have to beg people to come to meetings or to provide ideas or input.”
Sharing results	“I do think having it reported…through the [PACT Steering] committee as well as to the VISN, to the director, and they have their name on it as a work group and it was successful, it really just breeds more interest for the next project. And letting people know that it’s not one person that’s responsible for everything and that is a team effort, I think has been valuable.”
Barriers	
No pre-existing guidelines	“I think the hard parts were trying to figure out…out of the 700 people on the panel, who do we select? That’s been a process and it’s still an ongoing process”
Limited access to centralized data	“One of the challenges was getting everyone to understand the system: we didn’t have a place to store the data, no centralized database. [The data] wasn’t easy to share.”

**Table 2:** Outcomes of Adapting EBQI to Clarksburg Women’s Health Clinic Context

EBQI	Contextual factors	Outcomes
Multi-level stakeholder engagement	Prior QI project experience	Knowledge of site resources and gaps Inter-departmental communication Expanded support networks Accountability Confidence
Data-driven progress-monitoring	Limited access to data information systems Collaborative decision-making Flexibility	Development of site-specific tools Goal achievement
PDSA cycles	Time Team champion QI culture	Organizational learning Inter-departmental coordination “Fit-for-purpose” innovations
Sharing results	Prior QI project experience Time QI fair	Accountability Confidence “Spread” Expanded support networks

### Project Development

As an intervention site, the Clarksburg WHC team developed a roadmap of QI priorities with input from VISN-level stakeholders and members of PACT Steering Committee—a group of providers, project managers, and others at the Clarksburg VA who met monthly to assess PACT needs and progress. Addressing women Veterans’ mental health needs emerged as a clinic priority due to the high rates of mental health diagnoses among female patients (56% of panel enrollees had at least one mental health condition) and an increasing number of patients arriving at the clinic in an acute mental health crisis. However, the site decided to develop their first QI project around improving appointment scheduling for women Veterans seeking comprehensive primary care, as they had high confidence in their ability to complete this project without unanticipated hurdles. To improve appointment scheduling, the Clarksburg WHC team identified specific goals, developed a data collection plan, and collaborated with new departments to redesign the patient appointment system. By the end of the project period, they had met their QI goals.

This initial QI project allowed the Clarksburg team to learn new skills, identify new resources, and establish communication both with the research team and with other departments at their facility, which gave structure to the QI process (Table 1). The team also established valuable relationships with primary care and systems redesign. At the end of the project, the clinic initiated a QI fair to present their work and received an award in innovation from leadership, which significantly boosted the team’s confidence (Table 1). The clinic then developed a second QI project that aimed to enhance mental health services for women Veterans receiving primary care through WH-PACT.

### Progress-Monitoring

The team champion met weekly with the PCMHI team, nursing supervisors, and mental health supervisors to identify specific, manageable change targets. In calls with the research team, the Clarksburg team noted that as many as 30% of their newly enrolled female patients arrived in mental health distress. With support from the research team, the team thus decided on a two-pronged approach to improving care that included (1) prevention for newly enrolled patients, and (2) crisis management for existing patients and walk-ins. For newly enrolled WH-PACT patients, the Clarksburg WHC developed a process to identify patients’ emergent mental health needs by pre-screening patients for depression or other mental health conditions. For existing panel enrollees, the team developed a list of criteria to identify “high-risk” patients who may need more frequent monitoring and/or follow-up for appointments and medication refills. Finally, the Clarksburg WHC aimed to strengthen collaboration with mental health providers and improve the process of escorting women in crisis to the emergency room (ER), the mental health department, or other appropriate services—a process known as the “warm handoff.”

In the first 6 months of project development, the Clarksburg WHC identified and refined these goals, identified possible measures to track progress (including education outcomes, process changes, and prevalence data on patients’ mental and physical health), and had regular calls with the GLA team, who sent suicidality resources, assessment tools, and educational materials to support the QI project.

To achieve their first goal (identifying the emergent mental health needs of newly enrolled patients), the Clarksburg WHC adapted an existing tool the PCMHI team had used to pre-screen patients for depression or other mental health conditions. The Clarksburg PCMHI Registered Nurse (RN) conducted an initial assessment over the phone and determined whether patients needed referrals to mental health or more intensive services, such as case management. By calling patients before their first visit, the PCMHI RN was able to reach those patients who felt overwhelmed navigating the VA system and make emergency contact with patients who needed same-day or next-day appointments.

Achieving their second goal (creating a list of high-risk patients who needed additional monitoring for mental health conditions) proved more challenging. Without existing guidelines or a centralized database to filter through panel enrollees, the team champion felt uncertain about how to begin (Table 1). Ultimately, the team champion and the PCMHI RN conducted a literature review to identify appropriate diagnostic categories, then refined the criteria during team huddles while reviewing patient charts. The final list included standard diagnostic categories, such as current or past diagnoses of schizophrenia and PTSD and Care Assessment Needs (CAN) scores—a calculation of PACT patients’ probability of hospitalization or mortality^35^—as well as certain types of injuries, difficult life transitions, and direct referrals from the PCMHI team. In this way, the list was informed by both medical definitions and providers’ first-hand knowledge of patient needs.

To achieve their third goal (strengthening their relationship with mental health providers to improve the “warm handoff” process), the team champion established regular information-sharing sessions with individuals from different departments. During these meetings, the women’s clinic and mental health providers collectively developed a protocol for responding to patients in immediate crises, including outlining the responsibilities of a designated mental health contact. Meeting in person helped individuals with diverse expertise stay aligned to achieve shared goals (Table 1).

### Making Adjustments

Throughout implementation, PACT team members met weekly for 1 h to identify operational challenges, brainstorm potential solutions, and propose necessary adjustments to clinic practice—an approach modeled on the “Plan-Do-Study-Act” (PDSA) cycles. These adjustments were developed iteratively and tested on a small scale. For example, when escorting patients to the ER during the “warm handoff” process, the Clarksburg WHC nurses were concerned about publicly identifying patients as having “suicide ideation” or other specific mental health conditions. They therefore developed an ER card system that could be handed directly to ER staff without the risk of violating patients’ privacy.

Another adjustment emerged from the team’s on-going review of appointment data. Reports showed that 70% of “high-risk” patients did not follow through with referrals and appointments made to mental health. To address this gap, the Clarksburg WHC began to offer mental health services within the clinic; however, this innovation had little effect on appointment attendance, which providers attributed to stigma. To increase the likelihood of engagement with mental health services, the PCMHI RN decided to avoid using the term “mental health” directly when contacting high-risk patients over the phone, and referred instead to services that could assist with “big life changes” or “overwhelming stress.” However, the team did not measure whether this approach impacted appointment attendance.

### Sharing Results

Throughout the QI project, the Clarksburg team shared their progress and results with VISN leadership and the PACT Steering Committee, as well as internally with inter-disciplinary team members to facilitate problem-solving. One of the Clarksburg WHC nurses was a member of the facility-wide PACT Champion group and was able to share questions and concerns about their project directly with the larger PACT network as well as communicate new initiatives or opportunities in Women’s Health back to the clinic (Table 1). The team champion also felt supported by other departments within the facility.

At the end of the QI project period, the Clarksburg WHC had successfully achieved all three goals, including completing baseline assessments and connecting patients to appropriate services, developing criteria to identify high-risk patients, and strengthening their partnership with mental health. In addition to tracking the numbers of patients who were connected to appropriate mental health resources, the clinic observed high levels of satisfaction among providers and staff following successful implementation, which generated pride and allowed them to spread their ideas (Table 1).

## DISCUSSION

The Clarksburg WHC applied core elements of EBQI to improve the quality of health services for women Veterans with complex mental health needs. These elements were applied within a specific clinic context in ways that facilitated or impeded EBQI success at different moments throughout implementation. Aspects of clinic context that facilitated success include prior QI experience, protected time for providers, and the characteristics of individual team members, while limited access to a centralized data system posed a barrier to implementation. Through the interaction of EBQI elements and clinic context, the Clarksburg WHC accomplished its QI goals, strengthened inter-disciplinary partnerships, and bolstered team confidence.

### Multi-Level Stakeholder Engagement

The Clarksburg WHC drew support from a range of stakeholders, including VISN leadership, the research team, and other facility partners. Multi-level stakeholder engagement is a core feature of EBQI that promotes meaningful research-clinical partnerships and effective, bi-directional communication to increase the likelihood of QI success.^17,36^ Engaging multi-level stakeholders early on in the implementation process may be particularly valuable to secure buy-in and inspire confidence in the QI team.^34^ For the Clarksburg WHC, having early support from senior leadership bred accountability and confidence in individual team members, while support from the GLA team, such as through weekly check-ins, brought much-needed structure to the initial stages of project development. Internal partnerships between the Clarksburg WHC and mental health, systems design, and primary care fostered inter-departmental communication and coordination throughout the QI process, which helped prevent siloing.^34,37^

These multi-level partnerships were strengthened by the clinic’s prior experience conducting a QI project with GLA facilitation. Prior QI experience can generate awareness of a site’s strengths and limitations and build team members’ skills and confidence in their ability to develop and execute a QI intervention.^38,39^ In completing their first QI project, the Clarksburg team gained familiarity with practices such as scrubbing schedules, tracking no-show rates, contacting patients, and communicating with departments outside of the clinic to improve care—all of which proved valuable in completing their second QI initiative. Further, because the clinic was able to address foundational issues (e.g., staffing, appointment scheduling) during their first QI project, the clinic was better positioned to address improvements needed in mental health—a challenging area for implementing organizational change.^40^ Addressing foundational issues before embarking on more challenging QI initiatives may facilitate implementation success.^20,39^

### Tracking and Monitoring Progress

EBQI promotes data-driven problem-solving through the thoughtful selection of specific aims and measures and continuous surveillance of results.^20,34^ In clinic settings, access to data information systems and analysis of organizational data are positively associated with QI success.^28,41^ With research and technical assistance from GLA, the Clarksburg WHC developed manageable change targets by breaking their project into three distinct goals and identified appropriate techniques for tracking performance, such as the number of high-risk patients contacted and referrals to mental health services. However, unclear guidelines for identifying “high-risk” patients, as well as the lack of a centralized database to assist with the selection of such patients, presented potential roadblocks to progress-monitoring.

The Clarksburg team’s commitment to collective decision-making helped address these infrastructural gaps. Team huddles and regular check-ins provided an alternative mechanism for team members to keep track of evolving patients’ needs and clinic practices. In-person meetings also allowed team members to delegate responsibilities and develop new tools, such as a protocol for responding to patients arriving in acute crisis.

### PDSA Cycles

EBQI promotes the use of PDSA cycles, rooted in iterative, small-scale testing and rapid assessment of results to expedite improvement.^42^ While PDSAs can increase team members’ motivation, enhance problem-solving, and produce more robust, user-friendly changes,^43,44^ their success within complex healthcare systems varies widely depending on clinic resources, training capacity, and individual team members’ commitment to change efficacy.^45,46^

The Clarksburg WHC’s use of PDSA cycles was enhanced by both the team’s ability to commit to weekly meetings and advocacy from the team champion.^47^ Having protected time to invest in team-based reflection, systematic data surveillance, collective decision-making, or other QI activities has been shown to facilitate QI engagement.^34,48^ Team champions can facilitate QI success by harnessing team members’ strengths and individual interests and cultivating a QI culture conducive to experimentation and inter-departmental collaboration.^38,49,50^ While the Clarksburg team champion initiated information-sharing sessions across departments, she also encouraged team members to remain attentive throughout the “do phase” and integrate observations into the subsequent study phase of PDSA.^46^ The Clarksburg team members’ observations about adverse patient experiences during the warm handoff process and persistent stigma around scheduling mental health appointments led to novel improvements (the ER card system and co-located mental health care, respectively) that were developed rapidly in response to emergent needs and discontinued when proven ineffective or no longer useful. With protected time and supportive leadership, the Clarksburg WHC effectively used PDSA cycles to respond to challenges and identify “fit-for-purpose” solutions that fostered experimentation and minimized risk to patients.^39,42^

### Sharing Results

The Clarksburg team’s commitment to sharing updates on their progress not only kept team members accountable but also expanded their network of support and strengthened facility-wide communication such that team members knew whom to contact when problems arose. Communicating results, work practices, and improvement processes facilitates organizational learning and is an important component of spread.^51^ The Clarksburg WHC had established the practice of sharing results during their first QI initiative; their participation in a QI fair generated broader recognition of the team’s capabilities and helped secured buy-in from senior leadership, which is critical for the development of future QI innovations.^20,28^ Executive-level support may be particularly important for QI initiatives focused on women’s primary care.^34^

## CONCLUSIONS

EBQI offers valuable tools for clinics to improve integrated primary care and mental health services for women Veterans through the development of, and experimentation with, incremental changes to clinic practice. The process of developing an EBQI innovation may also expand support networks, enhance the skills and capabilities of individual team members, and lead to practices of inter-departmental collaboration and communication that become seamlessly integrated into daily clinic operations.

Implementation success results from the interplay of evidence-based methods and contextual factors, including access to resources, supportive infrastructure and leadership, and an organizational culture responsive to innovation and improvement.^48,52–55^ Because clinic resources, team members, policies, and QI culture are likely to shift over time, sites may benefit from on-going documentation of contextual factors and assessment of their associations with implementation outcomes.^56^ Mapping out the ways in which clinic context can support or impede EBQI adaptation may prove valuable as an on-going exercise for sites to identify areas where additional support is needed to facilitate QI success.

## Data Availability

The datasets generated and analyzed during the current study are not publicly available due to the inclusion of potentially sensitive disclosures, potential for subject re-identification, and lack of consent for data sharing from study participants.
